# Anxiety and Depression in Metabolic-Dysfunction-Associated Fatty Liver Disease and Cardiovascular Risk

**DOI:** 10.3390/jcm11092488

**Published:** 2022-04-28

**Authors:** Abdulrahman Ismaiel, Mihail Spinu, Daniel-Corneliu Leucuta, Stefan-Lucian Popa, Bogdan Augustin Chis, Mihaela Fadgyas Stanculete, Dan Mircea Olinic, Dan L. Dumitrascu

**Affiliations:** 1 2nd Department of Internal Medicine, “Iuliu Hatieganu” University of Medicine and Pharmacy, 400006 Cluj-Napoca, Romania; abdulrahman.ismaiel@yahoo.com (A.I.); popa.stefan@umfcluj.ro (S.-L.P.); bogdan_a_chis@yahoo.com (B.A.C.); ddumitrascu@umfcluj.ro (D.L.D.); 2 Medical Clinic No. 1, “Iuliu Hatieganu” University of Medicine and Pharmacy, 400006 Cluj-Napoca, Romania; spinu_mihai@yahoo.com (M.S.); danolinic@gmail.com (D.M.O.); 3 Department of Medical Informatics and Biostatistics, “Iuliu Hatieganu” University of Medicine and Pharmacy, 400349 Cluj-Napoca, Romania; dleucuta@umfcluj.ro; 4 Department of Neurosciences, “Iuliu Hatieganu” University of Medicine and Pharmacy, 400012 Cluj-Napoca, Romania; 5 Institute of Advanced Studies in Science and Technology, Babes-Bolyai University, 400084 Cluj-Napoca, Romania; 6 Interventional Cardiology Department, Emergency Clinical Hospital, 400006 Cluj-Napoca, Romania

**Keywords:** metabolic-dysfunction-associated fatty liver disease (MAFLD), hepatic steatosis, SteatoTest, anxiety, depression, Beck depression inventory, cardiovascular disease

## Abstract

(1) Background: The relationship between anxiety and depression in metabolic-dysfunction-associated fatty liver disease (MAFLD) and cardiovascular (CV) risk remains uncertain. Therefore, we aimed to assess whether anxiety and depression are associated with increased CV risk in MAFLD. (2) Methods: We conducted a cross-sectional observational study involving 77 subjects (39 MAFLD patients, 38 controls), between January and September 2020. Hepatic steatosis was assessed using a combination of hepatic ultrasonography and SteatoTest^TM^. CV parameters were evaluated using echocardiography and Doppler ultrasound. Self-reported questionnaires pertaining to symptoms of anxiety and depression were used. Anxiety was evaluated using Lehrer Woolfolk Anxiety Symptom Questionnaire (LWASQ), further divided into somatic, behavioral, and cognitive factors, as well as a global score, and depression using Beck Depression Inventory (BDI). (3) Results: MAFLD patients presented significantly higher BDI scores (*p*-value 0.009) and LWASQ global scores (*p*-value 0.045) than controls. LWASQ somatic factor was significantly associated with global longitudinal strain (GLS) in linear analysis (−0.0404, *p*-value = 0.002), while it lost significance following multivariate analysis (−0.0166, *p*-value = 0.124). Although group (MAFLD vs. controls) predicted BDI, LWASQ global score, and LWASQ somatic factor in linear regression, they lost significance in multivariate analysis. Moreover, the relationship between interventricular septal wall thickness (IVSWT) and BDI, LWASQ global score, and LWASQ somatic factor was significant in linear analysis, but statistical significance disappeared after multivariate analysis. (4) Conclusions: Although MAFLD patients presented increased anxiety and depression risk in univariate analysis, this association lost significance in multivariate analysis. A significant association between GLS levels and LWASQ somatic factor, in addition to IVSWT in anxiety and depression in univariate analysis, was observed, but was lost after multivariate analysis.

## 1. Introduction

Globally, the prevalence of nonalcoholic fatty liver disease (NAFLD) is rapidly increasing reaching up to 20–30% and is currently the most common chronic liver disease [1]. It is expected that NAFLD will become the most common indication for liver transplantation within the next decade [2]. The term metabolic-dysfunction-associated fatty liver disease (MAFLD) was lately proposed to replace the previously known nonalcoholic fatty liver disease (NAFLD) [3,4]. Because of the discrepancies in diagnostic criteria, the terms NAFLD and MAFLD should not be used interchangeably. MAFLD is commonly associated with multiple metabolic dysregulations, including cardiovascular disease (CVD) [5,6]. No pharmacological treatment for fatty liver disease has been approved yet, while the mainstay management remains to be lifestyle modification with weight loss [7]. Hence, it is essential to identify modifiable risk factors for MAFLD.

Anxiety disorders are the most common type of mental illness in the European Union, with a 12-month prevalence of up to 20% in adults, being more common than any other mental illness in European adults aged between 14 and 65, with the female sex being affected 2–3 times more frequently compared to males [8,9,10]. On the other hand, depression is the world’s third leading cause of disability, with a lifetime prevalence of major depressive episodes estimated to be between 3–29.9% [11,12]. It is recurrent and can lead to a decreased quality of life, disability, and mortality, being associated with a high burden for patients and imposing a significant impact on the healthcare systems [13,14].

While the relationship between anxiety and depression in NAFLD has been evaluated in several published studies, results have been conflicting [15,16,17,18,19,20,21]. In most cases, hepatic steatosis is a condition that is usually asymptomatic, hence, possibly leading to less mental health involvement. Anxiety disorders were observed in 7.9% of NAFLD patients, who were found to be significantly associated with state anxiety and trait anxiety in females, in addition to a higher risk in younger subjects [19,21]. It was observed that female NAFLD patients tend to present a higher risk of depression with increased liver fat deposition [19]. A recently published meta-analysis concluded that NAFLD patients had a higher prevalence of depression. This association was significantly higher in non-alcoholic steatohepatitis (NASH) patients than NAFLD patients [22]. However, the association between anxiety and depression in MAFLD using the recently defined diagnosis criteria has yet to be studied.

Emerging evidence suggests that anxiety and depression are linked to an increased risk of a variety of metabolic disorders, such as obesity, metabolic syndrome, and diabetes mellitus type 2, in addition to CVD, including hypertension, coronary artery disease, and stroke [23,24,25,26,27]. Nevertheless, the reverse pathway is also possible, where anxiety and depression can increase the risk of future metabolic disorders. Hence, a bi-directional relationship can be described. NAFLD, depression, and anxiety were found to share common risk factors, such as obesity and diabetes mellitus type 2 [28,29,30,31]. These findings suggest shared pathophysiological pathways, where inflammation in the central and peripheral immune system may connect metabolic syndrome with major depressive disorder, causing metabolic inflammation, which partly originates in the liver, in the central and peripheral immune system [32]. Moreover, insulin resistance was also suggested to exert an important role in this shared pathogenesis [33].

Although CVD has been associated with MAFLD, the exact pathophysiological mechanisms have not been clearly elucidated. Moreover, the current literature lacks data regarding anxiety and depression in MAFLD patients using the newly defined diagnosis criteria. Therefore, we hypothesized that anxiety and depression could increase the CV risk in MAFLD patients. Accordingly, we conducted an observational cross-sectional study assessing anxiety and depression in MAFLD patients vs. controls, in addition to multiple associated echocardiographic and Doppler ultrasound cardiovascular parameters.

## 2. Materials and Methods

### 2.1. Study Design, Setting, and Participants

This study was approved by the local ethical and research committee of “Iuliu Hatieganu” University of Medicine and Pharmacy Cluj-Napoca, (No. 486/21.11.2019). All procedures followed were in accordance with the ethical standards of the responsible committee on human experimentation (institutional and national) and with the Helsinki Declaration of 1975, as revised in 2008. All participants were informed regarding their ability to withdraw freely anytime from the study. The study design was explained to all participants, who provided an informed consent for participation before starting the study. The recruitment process, general definitions, blood tests, abdominal ultrasound, echocardiography, non-invasive markers, and scores have been previously discussed in more details [34,35].

In summary, this cross-sectional observational study investigated whether anxiety and depression could lead to increased CV risk in MAFLD. Moreover, we also evaluated how anxiety and depression are associated with the newly defined MAFLD. The study was conducted between January 2020 and September 2020 in the Clinical Emergency County Hospital of Cluj-Napoca, Romania. A non-probability consecutive sampling of eligible subjects was used. Eligible participants were males and females aged ≥18 and <65 years. All patients included in the MAFLD group had to fulfill the diagnosis criteria of MAFLD [36]. Hepatic steatosis diagnosis was based on ultrasonographic detection of fat deposition in the liver and SteatoTest^TM^ (BioPredictive, Paris, France). Patients were required to have hepatic steatosis using both ultrasonography and the SteatoTest^TM^ (BioPredictive) in order to be included in the MAFLD group, or else they were excluded. The subjects in the control group were mostly healthy hospital staff who did not meet the diagnostic criteria for MAFLD and did not have hepatic steatosis. Subjects with other secondary causes of hepatic steatosis, hepatitis B or C virus infections, liver tumors of malignant or benign etiologies, other coexistent liver diseases, in addition to acute inflammatory conditions such as deep venous thrombosis, systemic lupus erythematosus, active cancer, or history of any malignancies, active pulmonary exacerbations such as COPD exacerbation or asthma, acute infections (pulmonary, urinary, dental, COVID-19, etc.), failing to fast for at least 12 h prior to blood sampling, and refusing participation in the study were excluded. Medications including anxiolytics and antidepressants were reported after referring to the patients’ medical reports and documents. Moreover, chronic conditions were also evaluated and reported from the patients’ medical reports and documents.

### 2.2. General Definitions, Alcohol Consumption and Nicotine Dependence Assessment

#### 2.2.1. General Definitions

MAFLD diagnosis required the presence of hepatic steatosis in addition to one of the following three criteria: overweight/obesity, type 2 diabetes mellitus (DM), or confirmed metabolic risk dysregulations [36]. Hypertension was defined according to the 2020 International Society of Hypertension Global Hypertension Practice Guidelines [37]. The American Diabetes Association recommendations—Classification and Diagnosis of Diabetes: Standards of Medical Care in Diabetes—2021 were used to determine the diagnosis of diabetes and prediabetes [38]. The National Cholesterol Education Program guidelines were used to define dyslipidemia [39].

#### 2.2.2. CAGE Questionnaire

We used the Romanian version of the CAGE questionnaire to screen for alcohol abuse or misuse, which was completed by all enrolled subjects. This questionnaire has been reported to present a high test-retest reliability (0.80–0.95) and has been validated in multiple populations [40]. The CAGE questionnaire is composed of a four-item test with questions regarding alcohol intake Cutting down (C), Annoyed by criticism of other people about alcohol intake (A), feeling Guilty about alcohol intake (G), and using of Eye-openers morning drinking (E) [41]. A total of two or three affirmative responses were considered as a high level of suspicion for alcohol misuse/abuse. Scoring a 4 means the patient is likely to have alcoholism.

#### 2.2.3. The Alcohol Use Disorders Identification Test (AUDIT)

The Alcohol Use Disorders Identification Test (AUDIT) was completed by all participants, who were subsequently interviewed to clarify equivocal responses. In our study, we used the Romanian version of the AUDIT, a 10-item screening tool developed by the World Health Organization (WHO) [42], which defines one standard drink as 12 g of pure alcohol, while the Standard Drink chart was provided for clarifying such information. Possible answers on the AUDIT were used to evaluate specific drinking-related behavior during the preceding 12-month period, with scores ranging between 0 and 40, calculated by summing the scores of the evaluated ten items [43]. A score between 0–7 indicated low risk, 8–15 indicated increasing risk, 16–19 indicated higher risk, and ≥20 indicated possible dependence.

#### 2.2.4. The Fagerström Test for Nicotine Dependence (FTND)

Nicotine dependence was evaluated using the Fagerström Test for Nicotine Dependence (FTND), a reliable and validated tool used for the evaluation of smokers in different populations [44,45]. For the analysis, the FTND was scored as 1–2 points for low dependence, 3–4 points for low to moderate dependence, 5–7 points for moderate dependence, and ≥8 points for high dependence.

### 2.3. Evaluation of the State of Anxiety and Depression

#### 2.3.1. Beck Depression Inventory (BDI) Scale

Assessment of depression symptoms of included participants was performed using the Beck Depression Inventory (BDI) scale [46], a tool considered among the most used self-rating scales for measuring depression which has been validated in multiple populations (we used the Romanian version which was validated by David et al. with an internal consistency of 0.90) [47,48], composed of 21 items describing symptoms and attitudes related to depression. Each evaluated item is rated between 0 points (not at all) and 3 points (extreme form of each item). A sum of the obtained points composes the total score, ranging from 0 to 63, where a greater degree of depression is associated with a higher score. A score between 1–10 indicated normal ups and downs, 11–16 indicated mild mood disturbance, 17–20 indicated borderline clinical depression, 21–30 indicated moderate depression, 31–40 indicated severe depression, and >40 indicated extreme depression.

#### 2.3.2. Lehrer Woolfolk Anxiety Symptom Questionnaire (LWASQ)

The anxiety symptoms were evaluated using the Lehrer Woolfolk Anxiety Symptom Questionnaire (LWASQ), a tool that has been validated in several populations (we used the Romanian translation that has been validated by Mărginean et al.) [49,50,51]. It was demonstrated that the alpha Cronbach value for the somatic, behavioral and cognitive subscales were 0.92, 0.80, and 0.84, respectively [51]. Moreover, the overall scale score was reported to be 93, thus, indicating a high level of internal consistency [51]. This questionnaire includes a 36-item inventory covering somatic, behavioral, and cognitive aspects of anxiety. The somatic subscale assessment includes 16 items that refer to bodily symptoms related to anxiety. The behavioral assessment subscale includes nine items, mainly representing the evasion of social situations. The cognitive assessment subscale includes 11 items measuring the tendency to ruminate and worry. The score was divided into somatic, behavioral, and cognitive factors, and a global score including all items of the questionnaire.

### 2.4. Hepatic Ultrasonography

An experienced physician who was blinded to the study’s objectives, patients’ diagnoses, psychological measures, and lab results performed hepatic steatosis evaluation by ultrasonography using a GE LOGIQ S7 Expert [34,35]. A minimum of 8 h of fasting was required by participants before conducting the ultrasound evaluation. Subcostal and intercostal approaches were utilized to evaluate the liver parenchyma. The criteria used to assess hepatic steatosis included ultrasonographic contrast between the parenchyma of the liver and right kidney; the brightness of liver; evaluation of ultrasound deep attenuation penetration into the deep portion of the liver and altered diaphragmatic visualization; and altered visualization of intrahepatic vessels borders and narrowing of the lumen [52].

### 2.5. Cardiovascular Assessment

#### 2.5.1. Echocardiography

A board-certified cardiologist who was not aware of the study’s objectives, patients’ diagnoses, psychological measures, and labs performed a comprehensive echocardiographic and Doppler ultrasound evaluation using GE Vivid q Ultrasound Machine, as previously described [34,35], independent of the anxiety and depression assessment. We used the current recommendations and guidelines for the measurements and interpretations of the evaluated parameters [53,54,55,56,57,58]. These included M-mode, 2-dimensional, conventional color, and Doppler ultrasonography. Moreover, two-dimensional speckle-tracking echocardiography was utilized to calculate the Global Longitudinal Strain (GLS) and strain rate curves from all LV myocardial segments (4-chamber, 2-chamber, and long-axis apical views). We measured the average peak systolic longitudinal strain values and peak systolic strain rate.

#### 2.5.2. Electrocardiogram

Electrocardiogram (ECG) measurements and interpretations were performed by a physician blinded to the patients’ diagnosis, study aims, and labs. All enrolled subjects underwent a 12-lead electrocardiogram (ECG) assessment using 12 standard leads, the sensitivity of 10 mm/mV, and a recording speed of 25 mm/s. The ECG recordings were scanned and subsequently magnified in order to obtain several data, including heart rate (beats/min), RR interval (ms), PR interval (ms), QRS complex duration (ms), measured QT (QTm) interval (ms), corrected QT (QTc) interval (ms) calculated using the Bazett’s formula (QTcB = QTm/√RR) [59], measured JT (JTm) interval (ms), and corrected JT (JTc) interval (ms). The JTm was identified as the QT interval−QRS complex, and the corrected (JTc) was considered as QTcB−QRS complex [60].

### 2.6. Laboratory Analysis

The recommended protocols for blood sampling and analyzing the obtained blood samples through venipuncture were followed. All participants were required to fast overnight for a minimum of 12 h.

#### FibroMax

Ten serum biomarkers that are included in the FibroMax score were assayed from sera that were separated and stored at 2–8 °C for one day at most, while age, gender, weight, and height were adjusted to calculate the FibroMax score.

The serum levels of gamma-glutamyl transferase (GGT), total bilirubin, aspartate aminotransferase (AST), alanine aminotransferase (ALT), triglycerides, and total cholesterol were assessed using spectrophotometry (Atellica from Siemens), while haptoglobin, apolipoprotein A1, and α2-macroglobulin were evaluated using nephelometry (BN ProSpec System from Siemens). Plasma fasting glucose levels were measured using NaF/K2 oxalate spectrophotometry. The obtained values of the analyzed blood variables were entered into the BioPredictive network, where the computed algorithms were run.

In this investigation, SteatoTest was carried out in addition to abdominal ultrasonography to confirm hepatic steatosis. SteatoTest is a measure of the steatosis grade in hepatocytes that varies from S0 to S3 [61].

### 2.7. Statistical Analysis

The statistical analysis was performed using R software environment for statistical computing and graphics version 4.0.2 (R Foundation for Statistical Computing, Vienna, Austria). Categorical values were reported as frequencies and percentages. Normally distributed continuous values were reported as mean (standard deviation, SD), while non-normally distributed continuous values were reported as median (interquartile range, IQR). The *t*-test was utilized for normally distributed data of independent samples in order to compare the clinical characteristics of the study population as per the categorized groups. For non-normally distributed data, the Wilcoxon rank-sum test was utilized, while for categorical data, the chi-square test and Fisher exact test were utilized.

We conducted Spearman correlations in order to evaluate the association between anxiety and depression scores with MAFLD and several cardiovascular parameters. Moreover, univariate and multivariate linear regression models to control for confounding factors such as age (years), sex, group (MAFLD vs. controls), marital status and education standardized score, comorbidities at high anxiety risk score, body mass index, type 2 diabetes mellitus, systolic blood pressure, diastolic blood pressure, polypharmacy standardized score, anxiolytics, antidepressants, smoking dependence, and alcohol standardized score were conducted.

For all conducted linear models, we assessed the assumptions of residual normality by a quantile-quantile plot, heteroskedasticity using a standardized residual vs. fitted values, the presence of high leverage, high residuals, or high influential points using standardized residuals vs. hat-values vs. Cook’s distance plot, and the linearity relation of continuous variables with the outcome using component + residual plot. Furthermore, we assessed the presence of multicollinearity in multivariate models using variance inflation factors and correlation coefficients.

The regression results were reported as model coefficients, 95% confidence interval (CI—computed with robust variance sandwich estimators in case of heteroskedasticity), and *p*-value. For all conducted statistical analyses, two-sided statistical tests were performed. Statistical significance was considered with a *p*-value < 0.05.

## 3. Results

### 3.1. General Characteristics

**Figure 1:**
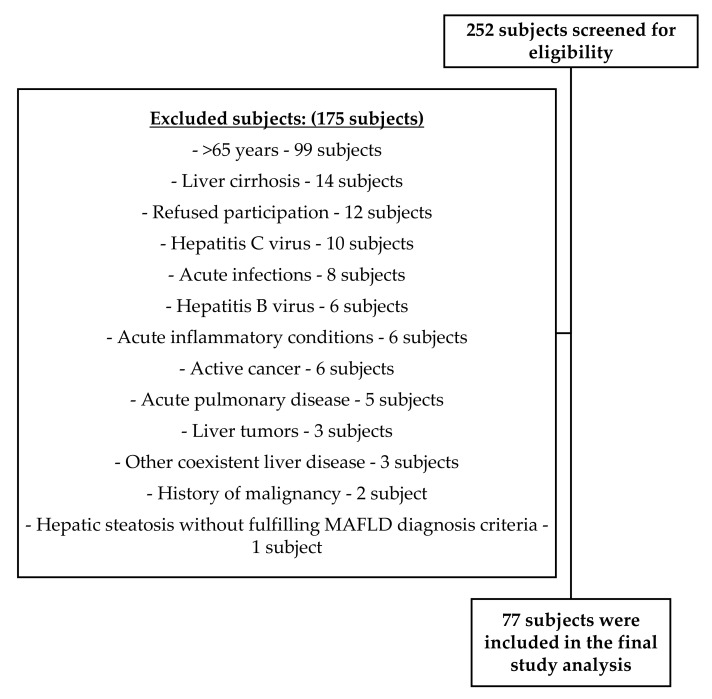
Flow diagram of included and excluded participants.

As demonstrated in Figure 1, a total of 252 subjects were screened for eligibility, out of which 175 subjects were excluded for the following reasons: 99 subjects were more than 65 years old, 14 subjects had liver cirrhosis, 12 subjects refused participation, 10 subjects presented hepatitis C virus, 8 subjects presented acute infections, 6 subjects presented hepatitis B virus, 6 subjects were diagnosed with acute inflammatory conditions, 6 subjects were active cancer patients, 5 subjects presented acute pulmonary disease, 3 subjects presented liver tumors, 3 subjects were diagnosed with other coexistent liver diseases, 2 subjects presented a history of malignancy, 1 subject diagnosed with hepatic steatosis in the control group. The final study analysis included a total of 77 Caucasian individuals. In Table 1, we summarize the participants’ general characteristics.

Enrolled subjects were divided into MAFLD patients and controls. The MAFLD group included 39 patients (50.65%) and the controls 38 subjects (49.35%), with a total mean age of 46 (ranging from 30–56). Gender distribution was 42 females (54.55%) and 35 males (45.45%) (*p*-value = 0.901). A significant difference was reported regarding marital status, education, menopausal status, BMI, hepatic steatosis, SteatoTest, systolic blood pressure, diastolic blood pressure, and number of medications, with a *p*-value of <0.001. Regarding associated comorbidities, a significant difference was reported between both groups in type 2 diabetes mellitus and hypertension, with a *p*-value <0.001, as outlined in Table 2.

### 3.2. Evaluation of Anxiety, Depression, Alcohol Consumption, and Smoking

Table 3 summarizes the BDI scores, LWASQ scores, alcohol consumption, and nicotine dependence scores. A significant increase in the BDI score was reported in MAFLD patients compared with controls (*p*-value = 0.009). Moreover, regarding the LWASQ, a significantly higher score was observed in global score and somatic factor in MAFLD patients compared with controls. However, no significant association was reported in behavioral factor and cognitive factor scores. In regards to the prescription of anxiolytics and antidepressants, both groups were almost equal (*p*-value = 1).

Alcohol consumption was assessed using CAGE with a significantly higher score in MAFLD vs. controls (*p*-value = 0.007), and AUDIT with no significant difference observed (*p*-value = 0.091). Although a non-significant difference was observed in Fagerström smoking dependence score between both groups, smoking (pack/years) was significantly higher in MAFLD patients (*p* < 0.001).

### 3.3. Cardiovascular Assessment

Table S1 outlines the obtained echocardiographic and Doppler ultrasound parameters. MAFLD patients had a significantly lower LVEF, early diastolic velocity (a’), and early diastolic peak velocity (E), and a higher late diastolic peak velocity (A), E/e’ ratio, carotid intima media thickness (CIMT), ventricular diameters, septal wall thickness, left ventricular end systolic and end diastolic volumes, cardiac output, stroke volume, and left ventricular posterior wall thickness (LVPWT).

ECG findings are summarized in Table 4. No significant association regarding the ECG rhythm, heart rate, RR interval, QRS wave, QT interval, QTc interval, JTm, and JTc was found between both groups. However, a significantly increased P wave duration and PR interval were observed in MAFLD patients, compared to controls.

### 3.4. Association between Anxiety and Depression in MAFLD and Cardiovascular Risk

We performed univariate and multivariate linear regression models with several dependent variables including E/A ratio, average, BDI score, LWASQ global score and somatic factor, and interventricular septal wall thickness as outlined in Table 5. Diastolic blood pressure (DBP) was assessed as a predictor in E/A ratio with an unadjusted B of −0.0274 (95% CI −0.0351–−0.0197, *p*-value < 0.001) in univariate regression analysis. This association remained significant after multivariate regression analysis (−0.0139 [95% CI −0.0223–−0.0056, *p*-value = 0.002]). LWASQ somatic factor was evaluated as a predictor of average GLS in linear regression analysis with a significant association (−0.0404 [95% CI −0.0652–−0.0156, *p*-value = 0.002]), while significance was attenuated to non-significant levels after multivariate analysis (−0.0166 [95% CI −0.0375–0.0043, *p*-value = 0.124]).

Although group (MAFLD vs. controls) predicted BDI (6.0378 [95% CI 1.9523–10.1233, *p*-value = 0.005]), LWASQ global score (27.5661 [95% CI 5.6326–49.4997, *p*-value = 0.016]), and LWASQ somatic factor (17.5088 [95% CI 5.9149–29.1026, *p*-value = 0.004]) in linear regression models, these associations lost significance in multivariate analysis (Table 5).

Moreover, we assessed BDI score (0.0633 [95% CI 0.0314–0.0952, *p*-value < 0.001]), LWASQ global score (0.0112 [95% CI 0.0045–0.018, *p*-value = 0.002]), and LWASQ somatic factor score (0.0223 [95% CI 0.0099–0.0346, *p*-value < 0.001]) as predictors of interventricular septal wall thickness in linear regression models with significant associations observed. However, statistical significance was lost after conducting multivariate regression analysis (Table 5).

## 4. Discussion

Several published studies evaluated the relationship between anxiety and depression in NAFLD patients [15,16,17,18,19,20,21,22], as well as cardiovascular disease [23,24,25,26,27]. However, the current literature lacks studies assessing the association between anxiety and depression in MAFLD patients. Furthermore, it was not evaluated whether anxiety and depression are associated with increased CV risk in MAFLD patients. Accordingly, to the best of our knowledge, we conducted the first observational study to assess the relationship between anxiety and depression in MAFLD patients using the recently defined diagnosis criteria and associated CV risk. We observed that MAFLD patients were significantly associated with an increased risk of anxiety (LWASQ global score and somatic factor) and depression (BDI) in univariate analysis, while the significance was lost after performing multivariate regression analysis. Although average GLS was significantly predicted by the somatic factor of the LWASQ, and interventricular septal wall thickness was significantly predicted by BDI score, as well as LWASQ global score and somatic factor in univariate analysis, the association lost significance after multivariate linear regression analysis.

In this study, the association between MAFLD with anxiety, assessed using the LWASQ global score and somatic factor, and depression evaluated using the BDI, was significant in univariate analysis. However, the significance was lost after adjusting for confounding factors. Anxiety and depression are related with higher risk for metabolic disorders, with a possible reverse pathway that leads to increase in future metabolic risk in patients with anxiety and depression. Moreover, “reaction to illness” leading to anxiety or depression could be another contributing factor [62]. However, as hepatic steatosis is a condition that usually remains asymptomatic in most cases, it might lead to less frequent mental health impairment. The current literature contains conflicting data regarding the increased risk of anxiety and depression in NAFLD. A recently published study conducted by Choi et al. involving 25,333 subjects reported similar findings regarding anxiety, where a significant association was observed in univariate analysis, but statistical significance was lost after adjusting for age, sex, diabetes, systolic and diastolic pressure, and smoking [19]. Moreover, they reported no significant association between the presence of NAFLD and depression. Several studies reported that NAFLD female patients tend to suffer from an increased risk of depression and anxiety [18,19,21,63]. On the other hand, Xiao et al. conducted a recently published meta-analysis concluding that depression is highly prevalent and associated with NAFLD, while NASH patients presented a significantly higher depression risk compared with NAFLD patients [22].

As expected, we reported an increased risk of obesity, metabolic syndrome, diabetes mellitus type 2, hypertension in MAFLD patients compared with controls. These findings are supported by the recently defined diagnosis criteria of MAFLD requiring the presence of metabolic dysregulation [36,64]. On the other hand, scarce data are currently available in the literature regarding cardiovascular assessment in MAFLD patients [65,66]. We found multiple significant associations related to cardiovascular systolic, subclinical systolic, and diastolic functions, as well as structural parameters in MAFLD patients vs. controls. Kim et al. recently published an interesting study reporting that although NAFLD patients were not found to present an increased all-cause mortality risk after adjusting for metabolic confounders, MAFLD patients presented an increased all-cause and cardiovascular mortality [5]. Therefore, highlighting the importance of evaluating cardiovascular risk factors in MAFLD patients.

In our study, we reported that anxiety (LWASQ—somatic factor) was associated with subclinical systolic dysfunction assessed using GLS in univariate analysis, but lost significance after multivariate analysis. Although the relationship between anxiety, depression, and increased cardiovascular risk has been evaluated in the literature, we found no published studies evaluating anxiety and depression in relation to subclinical systolic function evaluated by GLS. A hypothetical relationship between anxiety and depression with GLS might be through autonomic nervous system disturbances that are found in these diseases. Systolic function was assessed by Bekfani et al. in relation to anxiety and depression, reporting higher anxiety levels in heart failure with preserved ejection fraction (HFpEF) compared with heart failure with reduced ejection fraction (HFrEF) [67]. Moreover, female patients with HFpEF presented higher depression scores compared with control female subjects without HF. In a recently published systematic review and meta-analysis, the authors reported the global prevalence of any severity depression in HF patients to be 41.9%, while moderate to severe severity of depression was 28.1% [68]. Moreover, the prevalence of depression was found to be increased in females than in males. According to another systematic review and meta-analysis, depression was found to be a significant and independent predictor of all-cause mortality in patients with HF [69]. Nevertheless, anxiety did not appear to present a strong correlation.

Moreover, we evaluated several structural cardiovascular parameters, including interventricular septal wall thickness that was found to be significantly associated with anxiety (LWASQ global score and somatic factor) and depression in univariate analysis, but was attenuated to unsignificant levels after multivariate analysis. A study conducted by Vu et al. reported that increased LV mass index, LV mean wall thickness were associated with depressive symptoms after correcting for demographic variables [70]. However, all associations lost significance after performing further adjustments for lifestyle and CV risk factors. A meta-analysis conducted by Emdin et al. reported an increased risk of various cardiovascular events such as stroke, coronary heart disease, heart failure, and cardiovascular mortality with anxiety disorders [71]. Another meta-analysis conducted by Nicholson et al. observed a significantly increased risk of future coronary heart disease associated with depression [72]. However, the authors suggested that these results should be interpreted with caution due to incomplete and biased availability of adjustment for conventional risk factors and coronary disease severity. Nevertheless, causality of these associations remains unclear.

An interesting aspect in the evaluated topic would be the prism of shared risk factors between multiple pathologies, including MAFLD, diverticular disease, anxiety, and depression. This can possibly help in understanding the complex relationship between these pathologies that share multiple common risk factors. A recently published study conducted by Pantic et al. demonstrated that patients with colonic diverticulosis and concomitant hepatic steatosis had metabolic dysregulation components [73]. Moreover, the authors also reported that hepatic steatosis was found more frequently in more severe cases of colonic diverticulosis. Patients with diverticular disease were found to be associated with an increased risk of anxiety and depression, as well as CV risk [74,75].

We need to discuss several limitations in our study. Due to the modest sample size of our study, we were unable to conduct subgroup analyses based on participant gender and hepatic steatosis severity. We included Caucasian subjects of European background, therefore, generalization of the obtained results on other populations cannot be confirmed without further studies confirming our results. Due to the observational study design, we cannot confirm or negate causality regarding our findings. As a result, due to the complex connection with other comorbidities and the difficulties in determining causality, future studies using a longitudinal design may be beneficial in intending to resolve this challenge [76]. Furthermore, we used ultrasonography, reported to have a sensitivity ranging between 60–94% and specificity between 88–95% [77,78], in addition to SteatoTest^TM^ (Biopredictive) reported to have an AUROC of 0.81 [79,80], to evaluate and diagnose hepatic steatosis. Although liver biopsy remains the current gold standard for assessing hepatic steatosis, it is associated with several risks and raises additional ethical concerns in healthy individuals. As the controls were significantly younger, more educated, less likely to be married and to have cardiometabolic risk factors, possibly leading to less anxiety and depression in the control group compared to MAFLD patients, we corrected for such confounders using regression models.

Nevertheless, we also have several important strengths in our study. We improved the prediction accuracy for hepatic steatosis detection by combining both hepatic ultrasonography and SteatoTest^TM^ (Biopredictive). Moreover, we used the recently defined criteria for diagnosing MAFLD that was observed to identify patients with fatty liver disease, patients associated with higher risk for disease progression [64], and its association with anxiety and depression. Furthermore, we evaluated multiple cardiovascular parameters in MAFLD patients and their association with anxiety and depression. To the best of our knowledge, this is the first study to evaluate the association between anxiety and depression symptoms in MAFLD patients, and cardiovascular parameters.

## 5. Conclusions

Although MAFLD patients presented an increased risk for anxiety (assessed using Lehrer Woolfolk Anxiety Symptom Questionnaire) and depression (assessed using Beck depression inventory) in univariate analysis, this association was lost in multivariate regression analysis. Despite subclinical systolic dysfunction being observed in subjects with increased scores of somatic factor assessment in Lehrer Woolfolk Anxiety Symptom Questionnaire in univariate analysis, the significance was attenuated after adjustment using multivariate analysis. Furthermore, interventricular septal wall thickness was found to be associated with anxiety and depression. However, the statistical significance disappeared after multivariate analysis.

Future studies with larger sample sizes are required in order to confirm our obtained results and conduct subgroup analysis according to sex and hepatic steatosis severity in MAFLD patients. Moreover, further studies can possibly benefit from designs that are specifically developed to clarify causality between anxiety and depression in MAFLD patients and cardiovascular disease.

## Figures and Tables

**Table 1:** General characteristics of included participants.

Characteristic	Total (*n* = 77)	Control (*n* = 38)	MAFLD (*n* = 39)	*p*-Value
Age (years), median (IQR)	46 (30–56)	30 (27–41.5)	53 (48.5–58.5)	<0.001
Gender (male), *n* (%)	35 (45.45)	17 (44.74)	18 (46.15)	0.901
Marital Status:				<0.001
Single, *n* (%)	21 (27.27)	18 (47.37)	3 (7.69)	
Married, *n* (%)	51 (66.23)	19 (50)	32 (82.05)	
Divorced, *n* (%)	3 (3.9)	0 (0)	3 (7.69)	
Widow, *n* (%)	2 (2.6)	1 (2.63)	1 (2.56)	
Married (yes), *n* (%)	51 (66.23)	19 (50)	32 (82.05)	0.003
Education:				
Primary School, *n* (%)	3 (3.9)	0 (0)	3 (7.69)	<0.001
Middle School, *n* (%)	12 (15.58)	4 (10.53)	8 (20.51)	
High School, *n* (%)	27 (35.06)	7 (18.42)	20 (51.28)	
Undergraduate Course, *n* (%)	6 (7.79)	5 (13.16)	1 (2.56)	
University, *n* (%)	26 (33.77)	21 (55.26)	5 (12.82)	
Post-graduate, *n* (%)	3/77 (3.9)	1 (2.63)	2 (5.13)	
Menopausal status: *				<0.001
Premenopause	21 (50)	18 (42.86)	3 (7.14)	
Menopause	21 (50)	3 (7.14)	18 (42.86)	
Metabolic syndrome (yes), *n* (%)	32 (41.56)	2 (5.26)	30 (76.92)	<0.001
BMI, median (IQR)	26.57 (22.22–31.44)	22.18 (20.15–24.95)	31.18 (28.08–34.98)	<0.001
Obesity (yes), *n* (%)	22 (28.57)	1 (2.63)	21 (53.85)	<0.001
Hepatic steatosis (US) (yes), *n* (%)	39 (50.65)	0 (0)	39 (100)	<0.001
SteatoTest score, median (IQR)	0.39 (0.13–0.62)	0.13 (0.08–0.21)	0.62 (0.51–0.72)	<0.001
SBP-mean (mmHg), median (IQR)	124.5 (116–137)	120.75 (112.5–125.88)	133 (122.25–148.25)	<0.001
DBP-mean (mmHg), median (IQR)	79 (74–84)	75 (70.75–79)	83.5 (78.25–89)	<0.001
Number or medications, median (IQR)	1 (0–4)	0 (0–0)	3 (1.5–5.5)	<0.001

* Reported in 42 female subjects. BMI—Body mass index; DBP—Diastolic blood pressure; IQR—Interquartile range; MAFLD—Metabolic-associated fatty liver disease; SBP—Systolic blood pressure.

**Table 2:** Associated comorbidities of participants.

Characteristic	Total (*n* = 77)	Control (*n* = 38)	MAFLD (*n* = 39)	*p*-Value
Diabetes Mellitus Type 2 (yes), *n* (%)	15 (19.48)	0 (0)	15 (38.46)	<0.001
Impaired fasting glucose (yes), *n* (%)	5 (6.49)	2 (5.26)	3 (7.69)	>0.99
Hypertension (yes), *n* (%)	37 (48.05)	6 (15.79)	31 (79.49)	<0.001
Chronic kidney disease (yes), *n* (%)	1 (1.3)	0 (0)	1 (2.56)	>0.99
Ischemic heart disease (yes), *n* (%)	4 (5.19)	0 (0)	4 (10.26)	0.115
History of cerebrovascular accident (yes), *n* (%)	2 (2.6)	0 (0)	2 (5.13)	0.494
History of transient ischemic attack (yes), *n* (%)	1 (1.3)	0 (0)	1 (2.56)	>0.99
Irritable bowel syndrome (yes), *n* (%)	7 (9.09)	4 (10.53)	3 (7.69)	0.711
Gastroesophageal reflux disease (yes), *n* (%)	2 (2.6)	1 (2.63)	1 (2.56)	>0.99
Chronic pancreatitis (yes), *n* (%)	1 (1.3)	0 (0)	1 (2.56)	>0.99
Fibromyalgia (yes), *n* (%)	1 (1.3)	0 (0)	1 (2.56)	>0.99

MAFLD—Metabolic-associated fatty liver disease.

**Table 3:** Assessment of anxiety, depression, alcohol consumption, and smoking.

Characteristic	Total (*n* = 77)	Control (*n* = 38)	MAFLD (*n* = 39)	*p*-Value
BDI (21 questions), median (IQR)	10 (5–15)	8 (2–12.75)	12 (7–18)	0.009
LWASQ—Global Score, median (IQR)	91 (68–140)	85 (64.25–116)	100 (76.5–162.5)	0.045
LWASQ—Somatic factor, median (IQR)	35 (24–60)	27.5 (20.25–46.75)	47 (29–79.5)	0.003
LWASQ—Behavioral factor, median (IQR)	22 (18–33)	22.5 (17.25–26.75)	22 (18–37)	0.409
LWASQ—Cognitive factor, median (IQR)	34 (24–46)	31 (22–44)	38 (26.5–53.5)	0.128
Anxiolytics (yes), *n* (%)	5 (6.49)	2 (5.26)	3 (7.69)	>0.99
Antidepressants (yes), *n* (%)	3 (3.9)	1 (2.63)	2 (5.13)	>0.99
AUDIT, median (IQR)	1 (0–3)	2 (1–3)	1 (0–2.5)	0.091
CAGE, median (IQR)	0 (0–1)	0 (0–0)	0 (0–1)	0.007
Smoking history:				0.935
Smoker, *n* (%)	15 (19.48)	8 (21.05)	7 (17.95)	
Never smoked, *n* (%)	17 (22.08)	22 (57.89)	23 (58.97)	
Ex-smoker, *n* (%)	45 (58.44)	8 (21.05)	9 (23.08)	
Smoking (pack/years), median (IQR)	10 (6–16)	6 (2–8.5)	17 (10.75–20.5)	<0.001
Fagerström nicotine dependence score, median (IQR) *	4 (3–6.75)	4 (3.75–6.5)	4.5 (2.25–6.75)	0.845

* 7 MAFLD patients and 8 controls were active smokers and completed this questionnaire. AUDIT—Alcohol Use Disorders Identification Test; BDI—Beck depression inventory; CAGE—CAGE Alcohol Questionnaire; IQR—Interquartile range; LWASQ—Lehrer Woolfolk Anxiety Symptom Questionnaire; MAFLD—Metabolic-associated fatty liver disease.

**Table 4:** Assessed electrocardiogram parameters.

Characteristic	Total (*n* = 77)	Control (*n* = 38)	MAFLD (*n* = 39)	*p*-Value
ECG—rhythm:				0.52
Normal Sinus Rhythm, *n* (%)	65 (84.42)	34 (89.47)	31 (79.49)	
Tachycardia, *n* (%)	2 (2.6)	1 (2.63)	1 (2.56)	
Bradycardia, *n* (%)	10 (12.99)	3 (7.89)	7 (17.95)	
Heart rate (bpm), median (IQR)	71 (66–77)	70.5 (66–75.5)	71 (64–78)	0.787
RR interval (ms), median (IQR)	850 (782–920)	856.5 (834–922.75)	845 (761.5–918)	0.287
QRS (ms), median (IQR)	92 (86–98)	91 (86.5–95.75)	92 (86–102)	0.275
P (ms), median (IQR)	108 (100–120)	105 (98.5–113.5)	118 (102–124)	0.002
PR interval (ms), median (IQR)	156 (140–170)	149.5 (134.5–160)	161 (144–180)	0.008
QT interval (ms), median (IQR)	382 (370–398)	382 (374.5–395.5)	380 (369–400)	0.967
QTc interval (Bazett’s formula) (ms), median (IQR)	413.12 (403.33–429.76)	412.17 (402.74–423.71)	421 (405.4–439.52)	0.064
measured JT (JTm) (ms), median (IQR)	290 (280–314)	290.5 (282.5–312.5)	290 (276–311)	0.83
corrected JT (JTc) (ms), mean (SD)	324.58 (26.49)	321.11 (24.93)	327.97 (27.82)	0.258

IQR—Interquartile range; MAFLD—Metabolic-associated fatty liver disease.

**Table 5:** Univariate and multivariate linear regression models predicting DBP (mmHg), Group (MAFLD vs. Controls), Beck depression inventory (21 questions), ASQ—global score, and ASQ—somatic assessment, using several dependent variables.

Dependent Variable	Predictor	B Unadjusted	(95% CI)	*p*-Value	B Adjusted	(95% CI)	*p*-Value
E/A ratio	DBP (mmHg) *	−0.0274	(−0.0351−0.0197)	<0.001	−0.0139	(−0.0223–−0.0056)	0.002
GLS—Average	LWASQ—Somatic factor **	−0.0404	(−0.0652–−0.0156)	0.002	−0.0166	(−0.0375–0.0043)	0.124
BDI (21 questions)	Group (MAFLD vs. Controls) ***	6.0378	(1.9523–10.1233)	0.005	−1.5151	(−5.3772–2.3469)	0.445
LWASQ—Global Score	Group (MAFLD vs. Controls) ****	27.5661	(5.6326–49.4997)	0.016	−10.3165	(−36.2982–15.6651)	0.439
LWASQ—Somatic factor	Group (MAFLD vs. Controls) ****	17.5088	(5.9149–29.1026)	0.004	−7.3504	(−22.0228–7.322)	0.33
Interventricular septal wall thickness (mm)	Beck depression inventory (21 questions) **	0.0633	(0.0314–0.0952)	<0.001	0.021	(−0.024–0.0661)	0.364
Interventricular septal wall thickness (mm)	LWASQ—Global Score **	0.0112	(0.0045–0.018)	0.002	0.0041	(−0.003–0.0112)	0.266
Interventricular septal wall thickness (mm)	LWASQ—Somatic factor **	0.0223	(0.0099–0.0346)	<0.001	0.0055	(−0.0076–0.0187)	0.412

* Multivariate linear regression model adjusted for age (years), sex, group (MAFLD vs. controls), body mass index, type 2 diabetes mellitus, systolic blood pressure, Beck depression inventory (21 questions). ** Multivariate linear regression model adjusted for age (years), sex, group (MAFLD vs. controls), body mass index, type 2 diabetes mellitus, systolic blood pressure, diastolic blood pressure. *** Multivariate linear regression model adjusted for age (years), sex, group (MAFLD vs. controls), marital status and education standardized score, comorbidities at high depression risk score, polypharmacy standardized score, antidepressants, smoking dependence and alcohol standardized score. **** Multivariate linear regression model adjusted for age (years), sex, group (MAFLD vs. controls), marital status and education standardized score, comorbidities at high anxiety risk score, polypharmacy standardized score, anxiolytics, smoking dependence and alcohol standardized score. BDI—Beck depression inventory; CI—Confidence interval; DBP—Diastolic blood pressure; E/A ratio—Early diastolic peak velocity/Late diastolic peak velocity; GLS—Global longitudinal strain; LWASQ—Lehrer Woolfolk Anxiety Symptom Questionnaire; MAFLD—Metabolic-associated fatty liver disease.

## Data Availability

Data can be available upon request by contacting A.I. or S.-L.P.

## Supplementary Materials

The following supporting information can be downloaded at: https://www.mdpi.com/article/10.3390/jcm11092488/s1, Table S1: Evaluated cardiovascular ultrasound parameters.
