# Supplementary material for: Anxiety and Depression in Metabolic-Dysfunction-Associated Fatty Liver Disease and Cardiovascular Risk
Source: J Clin Med. 2022 Apr 28;11(9):2488. doi: 10.3390/jcm11092488 (PMC9102968; doi:10.3390/jcm11092488)
Supplement: Supplementary file 1 [file jcm-11-02488-s001.zip › jcm-1678991-supplementary.pdf]

Supplementary Table S1. Evaluated cardiovascular ultrasound parameters

**Characteristic**	**Total (*n*= 77)**	**Control (*n*= 38)**	**MAFLD (*n*= 39)**	**P-value**
CIMT - mean (mm), median (IQR)	8.5 (7 - 10)	7 (6.5 - 8.38)	10 (8.5 - 11)	< 0.001
Left atrial diameter (mm), mean (SD)	31.08 (4.64)	28.63 (4.1)	33.46 (3.84)	< 0.001
Left ventricular diameter (mm), median (IQR)	44 (40 - 47)	42 (38.25 - 44)	45 (43 - 49)	< 0.001
Right ventricular diameter (mm), median (IQR)	23 (21 - 26)	22 (20.25 - 24)	25 (22 - 27)	0.003
Interatrial septal wall thickness (mm), median (IQR)	6 (5 - 7)	5 (5 - 7)	6 (6 - 7)	0.013
Interventricular septal wall thickness (mm), median (IQR)	9 (8 - 10)	8 (8 - 9)	10 (9.5 - 11.5)	< 0.001
LVPWT (mm), median (IQR)	10 (8 - 10)	8 (8 - 9)	10 (10 - 11)	< 0.001
Early diastolic peak velocity - E (m/s), median (IQR)	0.73 (0.62 - 0.85)	0.8 (0.71 - 0.95)	0.65 (0.58 - 0.78)	< 0.001
Late diastolic peak velocity - A (m/s), median (IQR)	0.51 (0.43 - 0.73)	0.48 (0.42 - 0.56)	0.7 (0.49 - 0.8)	< 0.001
E/A ratio, median (IQR)	1.4 (1 - 1.8)	1.72 (1.33 - 1.98)	1.07 (0.73 - 1.42)	< 0.001
Early diastolic velocity - e' (m/s), median (IQR)	0.13 (0.11 - 0.17)	0.17 (0.14 - 0.2)	0.11 (0.08 - 0.13)	< 0.001
Late diastolic velocity - a' (m/s), median (IQR)	0.09 (0.07 - 0.14)	0.09 (0.07 - 0.13)	0.1 (0.08 - 0.16)	0.136
e'/a' ratio, median (IQR)	1.46 (0.88 - 2.14)	1.68 (1.44 - 2.4)	0.93 (0.69 - 1.61)	< 0.001
E/e' ratio, median (IQR)	5.37 (4.47 - 6.67)	5.05 (4.1 - 5.58)	5.92 (4.95 - 7.37)	0.004
LVEDV (ml), median (IQR)	95 (77 - 115)	84 (73.25 - 102)	103 (94 - 122.5)	< 0.001
LVESV (ml), median (IQR)	45 (37 - 56)	39 (32.75 - 47)	54 (44 - 63.5)	< 0.001
Ejection fraction (EF) (%), median (IQR)	50 (46 - 56)	52.5 (48 - 57)	48 (45.5 - 52)	0.004
Stroke volume (ml), median (IQR)	51 (39 - 57)	44 (36.25 - 56.5)	53 (46.5 - 57.5)	0.03
Cardiac output, median (IQR)	3.53 (2.88 - 4.33)	3.12 (2.61 - 3.96)	3.82 (3.09 - 5.13)	0.019
GLS – Long axis view (%), median (IQR)	20.2 (18.1 - 22.3)	21.5 (19.28 - 22.95)	18.9 (17.4 - 20.65)	< 0.001
GLS – 4-chamber view (%), median (IQR)	18.7 (17.2 - 20.6)	20.45 (18.35 - 21.87)	17.7 (15.95 - 19.1)	< 0.001
GLS – 2-chamber view (%), median (IQR)	18.2 (15.8 - 20.7)	20.3 (19.12 - 21.9)	16.1 (14.95 - 17.9)	< 0.001
GLS – Average (%), median (IQR)	19 (17.4 - 20.97)	20.92 (19.64 - 22.58)	17.67 (16.42 - 18.82)	< 0.001

GLS – Global longitudinal strain; IQR – Interquartile range; LVEDV – Left ventricular end diastolic volume; LVESV – Left ventricular end systolic volume; LVPWT – Left ventricular posterior wall thickness; MAFLD – Metabolic-associated fatty liver disease.
